# Helmet Use Among Two-Wheeler Riders’ Road Accident Victims in Benin

**DOI:** 10.1007/s44197-022-00077-x

**Published:** 2022-11-28

**Authors:** Bella Hounkpè Dos Santos, Alphonse Kpozehouen, Yolaine Glèlè Ahanhanzo, Donatien Daddah, Alain Levêque, Yves Coppieters

**Affiliations:** 1 Ecole de Santé Publique, Université Libre de Bruxelles, Brussels, Belgium; 2 Institut Régional de Santé Publique, Ouidah, Benin

**Keywords:** Helmet use, Trauma victims, Two-wheeler riders, Benin

## Abstract

**Background:**

In Benin, some riders of two-wheeled vehicles still do not wear helmets, the main protection against head injuries in road accidents. The objective of this study is to describe the characteristics of two-wheeled users, and to determine the factors influencing helmet use among this group.

**Methods:**

This is a prospective cross-sectional study of 977 two-wheeled road accident victims from a cohort. Proportions or means were calculated for the different variables. Statistical comparisons were made to test the association with helmet use. Logistic regression modelling was performed to identify factors associated with helmet use.

**Results:**

Among all subjects, 81.1% [CI_95%_ (78.5; 83.4)] wore a helmet. Factors explaining helmet use were female gender (OR = 2.8 [1.3–6.1]), purpose of trip (OR = 1.7 [1.1–2.6]), possession of health insurance (OR = 3.7 [1.3–10, 5]), having been driving for 15–20 years (OR = 2.6 [1.4–4.7]) or more than 20 years (OR = 3.4 [2.0–5.8]), good road conditions (OR = 3.1 [2.0–4.8]), and good visibility (OR = 1.9 [1.3–3.1]).

**Conclusion:**

The factors influencing helmet use are gender, reason for travel, length of time as a driver, possession of health insurance, conditions, and visibility of the road on which the subject are driving. These factors are related to experience and appropriation of the notion of risk, but also related to the environment. To increase helmet use among two-wheelers, helmet awareness should take into account the individual factors found in this study. Enforcement actions should be strengthened, and the quality of the roads improved.

## Introduction

Daily activities and various considerations lead people to use different types of roads with different means of transport that expose them to road accidents. Road accidents are responsible for 1.35 million deaths each year around the world [1]. Pedestrians and two-wheelers are the most vulnerable to road accidents, with motorcyclists accounting for 28% of deaths [1]. In Benin, motorcyclists are involved in more than half of all road traffic accidents and it is within this group that the majority of injuries and fatalities are recorded, according to statistics from the National Road Safety Centre [2].

One of the causes of road deaths among motorcyclists is head trauma [3]. To prevent these injuries, the WHO and the international community have recommended the use of helmets [1, 4]. Benin, adhering to the international initiative, adopted Decree N°72–113 of 27 April 1972 prescribing the compulsory wearing of helmets by drivers and passengers of two-wheeled vehicles or similar vehicles equipped with a thermal engine. The enforcement of this law was reinforced by the issuing of enforcement orders in the regions and checks on drivers.

Despite the existence of the law, compliance with this measure is not yet effective among all two-wheeled users in Benin. According to the authors, certain factors influence helmet use, including position on the motorbike and gender [5–8]. For Setty et al*.*, female drivers wear helmets more than men, but when men are passengers, they wear them more than women [5]. Age, purpose of travel, and the fact of having been stopped, ticked, or summoned by the police influence helmet use, according to some authors [5, 7, 9]. For example, people who commute to work or school wear helmets more compared to those travelling to/from leisure activities or travelling for pleasure [5]. Other factors for not wearing helmets mentioned by non-helmet wearers are the discomfort caused by helmets [6, 7, 10]. Time of day, day of the week, and weather conditions were also cited [6, 8]. Some users continue to forget to wear helmets, or wear them depending on the distance they travel or where they are going [7]. Availability and cost limit ownership and therefore use [11].

The aim of this study is to describe the characteristics of traffic accident victims with regard to helmet use and to determine the factors influencing helmet use in Benin.

## Methods

### Type of Study

This is a prospective cross-sectional, descriptive, and observational study with an analytical focus.

### Study Framework

The study was conducted in Benin, a country in West Africa, subdivided in twelve regions. The country’s health system has a pyramidal structure with three levels of health facilities/hospitals. In 2020, there were a total of eight hospitals of central level, six hospitals of intermediate level, and 30 hospitals of peripheral level. There were also 1079 others public and private health facilities. For the implementation of the cohort, three regions were selected. In 2020. These three regions notified 22,216 cases of trauma by road accident out of a total of 71,069 cases notified in the country [12]. In each of these regions, referral hospitals, that receive a large number of road traffic injuries according to data provided by the health information system, were retained making a total of five hospital. In the Littoral region (south of Benin), these were the National University Hospital Hubert Koutoukou Manga (CNHU-HKM) and the district hospital of Menontin. In Ouémé region (south of Benin), it was the Regional University Hospital of Ouémé (CHUDO), and in the Borgou region (north of Benin), the hospitals concerned were the Regional University Hospital of Borgou (CHUDB) and the district hospital of Boko.

In Benin, care is entirely the responsibility of the patient. Social security is almost non-existent. Barely 10% of people, with medium-to-high incomes and the notion of prevention of disease and risk, subscribe to insurance [13].

### Study Population and Sources

**Fig. 1:**
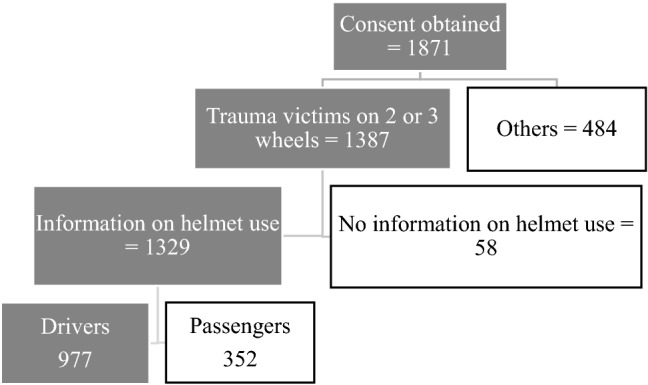
Selection process of subjects in the TraumAR cohort database

The study was conducted on a cohort of road traffic accident victims from July 2019 to January 2020. A total of 1871 consenting subjects were enrolled in this cohort named TraumAR, and 74.1% of these victims were on motorcycles. A consent for inclusion in the cohort was taken from each patient or a relative representing him. The data on the subjects in this cohort are general about the trauma victim or specific about the conditions of the accident and its setting, the subject's history and behaviour, and the details of clinical and paraclinical examinations. The subjects involved in the present study were all two-wheeled users enrolled in the TraumAR cohort, who were driving the motorbike at the time of the accident and for whom the helmet wearing status was recorded. Relevant data for the study were extracted from the TraumAR database (Fig. 1).

### Sampling and Sample Size

The sample was made up of all the subjects who were driving the motorbike during the accident and for whom the helmet wearing status was recorded, i.e., 977 subjects representing 73.6% of the two-wheeled users of the TraumAR cohort (Fig. 1).

### Variables Under Study

The main variable is the wearing of a helmet at the time of the accident. It is a dichotomous variable taking the values "yes" or "no" when the subject had his helmet on his head at the time of the accident. In connection with the main variable, we have researched the variable “having the safety strap closed at the time of the accident” and if not the reasons. The independent variables were selected, referring to factors found in the literature, to the data available in the TraumAR cohort, and empirically to findings in Benin. They were grouped into two sets of independent variables: those related to the individual and those related to the environment or vehicles. The first group includes gender (male or female), age in years, marital status (single, married, divorced), employment status (employed, in training, unemployed), reason for travel (private or professional), sector of activity (private, public/confessional), accident history, driving experience (in years), and insurance coverage (yes/no). The second group of variables includes the type of road, road condition, time of day, perceived level of visibility, and weather conditions.

### Data Processing and Analysis

The data were processed and analysed using STATA15 software. Pearson's Chi2 and Student's *t* tests were used at the 0.05 significance level for comparisons of proportions and means with respect to the conditions of use. A simple binary logistic regression and a top–down stepwise binary logistic regression model were performed to investigate the factors influencing helmet use. The variables included in the initial model were those with a *p* value less than or equal to 0.2. The variables retained in the final model were those with a *p* value of less than 0.05. The fit and specification of this model were checked.

## Results

### Characteristics of the Subjects

The average age of the two-wheeled users was 36.2 ± 12.5 years. Those not wearing helmets were younger (32.3 ± 12.3) than those wearing them (37.1 ± 12.3). Overall, 90.2% were male and 68.3% were married. About seven out of 10 injured people were in private travel at the time of the accident and 83.3% were employed. Of these, 88.7% were in private employment. Less than 10% of the injured two-wheelers had health insurance. In more than half of the cases, the accident occurred in a back street, and in full daylight in another half of cases. Approximately one in four of the injured riders considered the visibility to be good at the time of the accident, 77.8% said the road conditions were good and 80% said the weather conditions were good. They had been driving for an average of 16 years, and 35% had already had an accident (Table 1).

**Table 1:** Characteristics of motorbike users injured in road accidents according to helmet use, 2020, Benin

Factors	*n* (%) or *n* (mean ± SD)	Helmet use % or mean ± SD		Crude OR	*p* value
		Yes	No		
Gender					** < 0.001**
Female	96 (9.8)	90.6	9.4	2.4 (1.2–4.9)	
Male	881 (90.2)	80.0	20.0	1	
Age (years)	975 (36.2 ± 12.5)	37.1** ± **12.3	32.3** ± **12.3		** < 0.001**
< 20	51 (5.2)	62.8	37.2	1	
20–30	287 (29.4)	74.6	25.4	1.7 (0.9–3.3)	
30–40	294 (30.1)	83.3	16.7	3.0 (1.6–5.7)	
40–50	189 (19.4)	88.9	11.1	4.8 (2.3–9.8)	
> 50	154 (15.8)	85.7	14.3	3.6 (1.7–7.4	
Marital status					** < 0.001**
Single	281 (29.3)	73.0	27.0	1	
Divorced	25 (2.6)	96.0	4.0	8.9 (1.2–66.9)	
Married	654 (68.3)	84.1	15.9	2.0 (1.4–2.7)	
Employment status					**0.007**
Unemployed	32 (3.3)	87.5	12.5	2.8 (0.9–8.6)	
In employment	803 (83.3)	82.4	17.6	1.9 (1.2–2.9)	
In training	129 (13.4)	71.3	28.7	1	
Reason for travel					**0.003**
Private	675 (69.7)	78.7	21.3	1	
Business	293 (30.3)	86.7	13.3	1.8 (1.2 – 2.6)	
Sector of activity					**0.026**
Private	709 (88.7)	81.7	18.3	1	
Public or religious	90 (11.3)	91.1	8.9	2.3 (1.1–4.9)	
Accident history					**0.048**
Yes	322 (35.0)	85.4	14.6	1.4 (1.0–2.1)	
No	598 (65.0)	80.1	19.9	1	
Length of time in driving (years)	917 (16.1 ± 10.3)	16.9** ± **10.3	12.5** ± **9.4		** < 0.001**
< 8	191 (20.8)	72.8	27.2	1	
8–15	242 (26.4)	77.3	22.7	1.3 (0.8–2.0)	
15–20	175 (19.1)	86.9	13.1	2.5 (1.4–4.2)	
≥ 20	309 (33.7)	90.0	10.0	3.3 (2.1–5.5)	
Health insurance					** < 0.001**
Yes	98 (10.3)	94.9	5.1	4.8 (1.9–11.9)	
No	855 (89.7)	79.7	20.3	1	
Type of road					** < 0.001**
Interstate highways	151 (15.6)	74.2	25.8	1.8 (0.9–3.3)	
Rural tracks	58 (6.0)	62.1	37.9	1	
National Roads	223 (23.1)	78.9	21.1	2.3 (1.2–4.3)	
Lanes	535 (55.3)	85.8	14.2	3.7 (2.1–6.6)	
Pavement condition					** < 0.001**
Good	749 (77.8)	85.3	14.7	3.2 (2.2–4.6)	
Deteriorated	170 (17.7)	64.7	35.3	1	
Under construction	44 (4.6)	70.4	29.6	1.3 (0.6–2.7)	
Time of day					0.221
Dusk	1655 (16.9)	81.8	18.2	1.4 (0.8–2.2)	
Dawn	63 (6.5)	84.1	15.9	1.6 (0.8–3.4)	
Day	489 (50.2)	88.8	17.2	1.5 (1.0–2.1)	
Night	257 (26.4)	76.7	23.3	1	
Visibility					** < 0.001**
Medium	125 (12.9)	71.2	28.8	1	
Good	697 (71.7)	84.7	15.3	2.2 (1.4–3.4)	
Poor	150 (15.4)	73.3	26.7	1.1 (0.6–1.9)	
Climatic conditions					0.962
Good	80 (8.2)	81.0	19.0	1.0 (0.6–1.8)	
Poor	891 (91.8)	81.2	18.8	1	

### Helmet Use

Overall, 81.1% [CI_95%_ (78.5; 83.4)] were wearing helmets. A few of the subjects wearing helmets did not have the safety strap closed at the time of the accident (4.0%) and more than 80% of these declared that they had not closed it due to negligence. For 72.9% of the subjects not wearing a helmet, the helmet was either uncomfortable or would mess up their hair.

### Factors Associated with Helmet Use

Factors influencing helmet use in univariate analysis were gender, age, marital status, employment status, reason for travel, sector of employment, accident history, length of driving experience, insurance coverage, road type, road conditions, and visibility (Table 1).

Among the study subjects, women wore helmets twice as much as men (*p* < 0.001). As the age of the subjects increased, so did their helmet use. Thus, compared to subjects under 20 years of age, those aged 20–30, 30–40, 40–50, and over 50 years of age wore helmets 1.7, 3.0, 4.8, and 3.6 times more, respectively (*p* < 0.001). Married subjects protected themselves 2.0 and divorced subjects 8.9 times more than single subjects (*p* < 0.001). Employed users wore helmets 1.9 times more than those in training (*p* = 0.007), those travelling for work 1.8 (*p* = 0.003), and those working in the public or religious sector 2.3 (*p* = 0.026) times more than others. Those with a history of accidents wore helmets 1.4 times more than those who had never had a road accident (*p* = 0.048). The longer they had been driving, the more they wore helmets (*p* < 0.001). Subjects covered by health insurance wore their helmets 4.8 times more than those with no insurance (*p* < 0.001). It was observed that subjects wore helmets more when riding on good road conditions (*p* < 0.001) and when visibility was good (*p* < 0.001). Time of day and weather conditions did not influence helmet use.

In multivariate analysis, the factors explaining helmet use were gender, reason for travel, length of time as a driver, possession of health insurance, road conditions, and visibility.

Considering the other variables in the model, women wore helmets 2.8 times more than men; those who had their accident on a work trip wore them 1.8 times more than those who had their accident on a private trip. Adjusting for other variables, trauma victims who had been driving for 15–20 years wore helmets 2.6 times more, and those with more than 20 year experience wore them 3.4 times more than those with less than 8 year experience. Those with health insurance were 3.7 times more likely to wear a helmet than those without, and those riding on good roads were 3.1 times more likely to wear a helmet than those who had their accident on poor roads, adjusted for the other variables in the model. Taking into account gender, reason for travel, length of driving experience, possession of health insurance, and road condition, subjects wore helmets 1.9 times more when visibility was good than when it was poor (Table 2).

**Table 2:** Factors associated with helmet use among motorbike users injured in road accidents in multivariate analysis, 2020, Benin

Factors	OR adjusted (95% IC)	*p* value
Gender		0.011
Female	2.8 (1.3–6.1)	
Male	1	
Reason for travel		0.018
Private	1	
Business	1.7 (1.1–2.6)	
Length of time in driving (years)		< 0.001
< 8	1	
8–15	1.3 (0.8–2.1)	
15–20	2.6 (1.4–4.7)	
≥ 20	3.4 (2.0–5.8)	
Health insurance		0.016
Yes	3.7 (1.3–10.5)	
No	1	
Pavement condition		< 0.001
Good	3.1 (2.0–4.8)	
Deteriorated	1	
Under construction	1.1 (0.5–2.4)	
Visibility		0.038
Medium	1	
Good	1.9 (1.3–3.1)	
Poor	1.3 (0.7–2.5)	

## Discussion

The objective of the study was to describe the characteristics of traffic accident victims with regard to helmet use and to determine the factors influencing helmet use in Benin (Tables 3 and 4). The results showed that the proportion of subjects wearing helmets (81.1%) was close to that observed among two-wheeled users in South India (82.2%) and slightly lower than that of studies conducted in Malaysia (93.4%) and Tanzania (98.8%) [5, 8, 14]. All these studies are cross-sectional and directly observational of motorbike users in road traffic. In contrast, studies in Iran and Kenya among road traffic accident victims in hospitals show lower proportions of motorbike riders wearing helmets at the time of the accident (21.5% and 38.9%, respectively) [6, 10]. The latter results show that trauma victims were more likely to wear helmets in Benin.

**Table 3:** Characteristics of motorbike users injured in road accidents according to helmet use, 2020, Benin

Factors	*n* (%) or *n* (mean ± SD)	Helmet use % or mean ± SD		Crude OR	*p* value
		Yes	No		
Gender					** < 0.001**
Female	96 (9.8)	90.6	9.4	2.4 (1.2–4.9)	
Male	881 (90.2)	80.0	20.0	1	
Age (years)	975 (36.2 ± 12.5)	37.1 ± 12.3	32.3 ± 12.3		** < 0.001**
< 20	51 (5.2)	62.8	37.2	1	
20–30	287 (29.4)	74.6	25.4	1.7 (0.9–3.3)	
30–40	294 (30.1)	83.3	16.7	3.0 (1.6–5.7)	
40–50	189 (19.4)	88.9	11.1	4.8 (2.3–9.8)	
> 50	154 (15.8)	85.7	14.3	3.6 (1.7–7.4	
Marital status					** < 0.001**
Single	281 (29.3)	73.0	27.0	1	
Divorced	25 (2.6)	96.0	4.0	8.9 (1.2–66.9)	
Married	654 (68.3)	84.1	15.9	2.0 (1.4–2.7)	
Employment status					**0.007**
Unemployed	32 (3.3)	87.5	12..5	2.8 (0.9–8.6)	
In employment	803 (83.3)	82.4	17.6	1.9 (1.2–2.9)	
In training	129 (13.4)	71.3	28.7	1	
Reason for travel					**0.003**
Private	675 (69.7)	78.7	21.3	1	
Business	293 (30.3)	86.7	13.3	1.8 (1.2 – 2.6)	
Sector of activity					**0.026**
Private	709 (88.7)	81.7	18.3	1	
Public or religious	90 (11.3)	91.1	8.9	2.3 (1.1–4.9)	
Accident history					**0.048**
Yes	322 (35.0)	85.4	14.6	1.4 (1.0–2.1)	
No	598 (65.0)	80.1	19.9	1	
Length of time in driving (years)	917 (16.1 ± 10.3)	16.9** ± **10.3	12.5** ± **9.4		** < 0.001**
< 8	191 (20.8)	72.8	27.2	1	
8–15	242 (26.4)	77.3	22.7	1.3 (0.8–2.0)	
15–20	175 (19.1)	86.9	13.1	2.5 (1.4–4.2)	
≥ 20	309 (33.7)	90.0	10.0	3.3 (2.1–5.5)	
Health insurance					** < 0.001**
Yes	98 (10.3)	94.9	5.1	4.8 (1.9–11.9)	
No	855 (89.7)	79.7	20.3	1	
Type of road					** < 0.001**
Interstate highways	151 (15.6)	74.2	25.8	1.8 (0.9–3.3)	
Rural tracks	58 (6.0)	62.1	37.9	1	
National Roads	223 (23.1)	78.9	21.1	2.3 (1.2–4.3)	
Lanes	535 (55.3)	85.8	14.2	3.7 (2.1–6.6)	
Pavement condition					** < 0.001**
Good	749 (77.8)	85.3	14.7	3.2 (2.2–4.6)	
Deteriorated	170 (17.7)	64.7	35.3	1	
Under construction	44 (4.6)	70.4	29.6	1.3 (0.6–2.7)	
Time of day					0.221
Dusk	1655 (16.9)	81.8	18.2	1.4 (0.8–2.2)	
Dawn	63 (6.5)	84.1	15.9	1.6 (0.8–3.4)	
Day	489 (50.2)	88.8	17.2	1.5 (1.0–2.1)	
Night	257 (26.4)	76.7	23.3	1	
Visibility					** < 0.001**
Medium	125 (12.9)	71.2	28.8	1	
Good	697 (71.7)	84.7	15.3	2.2 (1.4–3.4)	
Poor	150 (15.4)	73.3	26.7	1.1 (0.6–1.9)	
Climatic conditions					0.962
Good	80 (8.2)	81.0	19.0	1.0 (0.6–1.8)	
Poor	891 (91.8)	81.2	18.8	1

**Table 4:** Factors associated with helmet use among motorbike users injured in road accidents in multivariate analysis, 2020, Benin

Factors	OR adjusted (95% IC)	*p* value
Gender		0.011
Female	2.8 (1.3–6.1)	
Male	1	
Reason for travel		0.018
Private	1	
Business	1.7 (1.1–2.6)	
Length of time in driving (years)		< 0.001
< 8	1	
8–15	1.3 (0.8–2.1)	
15–20	2.6 (1.4–4.7)	
≥ 20	3.4 (2.0–5.8)	
Health insurance		0.016
Yes	3.7 (1.3–10.5)	
No	1	
Pavement condition		< 0.001
Good	3.1 (2.0–4.8)	
Deteriorated	1	
Under construction	1.1 (0.5–2.4)	
Visibility		0.038
Medium	1	
Good	1.9 (1.3–3.1)	
Poor	1.3 (0.7–2.5)	

The proportion of subjects closing their helmet straps was higher in our study than in South India (35.3%) and Malaysia (40%) [5, 14]. It should be noted that these studies are direct observational studies, whereas the proportions in our study are based on self-reporting. Furthermore, in India, this proportion refers to subjects wearing helmets that comply with the country's standards [5]. The reasons for not wearing a helmet in the present study were also found in Kenya [6] and Iran [10].

Most of the factors associated with helmet use have been found by other authors [5, 9, 10, 15]. Indeed, in Malaysia, a study based on the Theory of Planned Behaviour using a multivariate analysis identified gender and the notion of a personal or family history of road accidents as factors associated with helmet use. The Malaysian study identified other factors not examined in the present study, such as the power of the motorbike, the notion of previous police control or sanction, and the attitude and perceived behaviour of others with regard to helmets [9]. The reason for travelling was included in the multivariate model in South India. These authors also noted the fact of having been checked by the police in the last 3 months, a factor not examined in our study [5]. Accident history was found in the model by Faryabi et al. This variable, although significant in univariate analysis, is not included in our final model [10]. Brown et al. found in their model that insurance ownership was associated with helmet use. This result is in line with ours, but, in contrast, they did not note gender. They included the position on the motorbike in their model, whereas we conducted our study only among drivers, as very few passengers wear helmets [15].

Some authors have observed that time of day, days of the week, and weather conditions may influence helmet use [6, 8]. Time of day and weather conditions were not significant in our study. Factors, such as visibility and road conditions, were in our model. Visibility can be influenced by time of day and weather conditions, among other things. Drivers wear helmets more on roads in good condition. In the national context, this observation could be explained by the fact that it is often major roads that are in good condition, and it is also on these roads that the risk of police controls is higher. Wearing a helmet on these roads would be to avoid sanction.

This study found that factors such as length of time driving and insurance coverage, which could be described as factors related to experience and knowledge of risk, contribute to helmet use in Benin. It also revealed the environmental conditions under which subjects are less likely to wear helmets. These observations could be used to reinforce behaviour change activities in specific groups. They should also lead to the involvement of subjects with some experience and knowledge of risk in the awareness and education of their peers.

## Conclusion

This study provided information on the profile of helmet wearers and the environmental conditions in which they are more likely to wear helmets. The factors found in this study that explained helmet use were gender, purpose of trip, factors related to experience, and appropriation of the notion of risk (driving experience, insurance coverage), but also factors related to the environment (conditions and visibility of the road on which subjects are riding). These must be considered in activities to improve helmet wearing and to target beneficiaries. The enforcement of the helmet law must be intensified on all types of roads day and night. Interventions to increase helmet wearing, for example public awareness and education activities need to be rethought and applied from a very young age to specific groups such as the self-employed, those in the private sector, those riding on bad roads, or those not covered by insurance, because these factors were associated with reduced helmet wearing among this sample of motorcycle riders. Road infrastructure development programs should be strengthened to improve road conditions and their lighting.
